# Comorbidities and Angiogenic Regulators Affect Endothelial Progenitor Cell Subtype Numbers in a Healthy Volunteer Control Group

**DOI:** 10.1007/s12015-024-10777-5

**Published:** 2024-08-26

**Authors:** Kamini Rakkar, Rais Reskiawan A. Kadir, Othman A. Othman, Nikola Sprigg, Philip M. Bath, Ulvi Bayraktutan

**Affiliations:** 1 Translational Medical Sciences, School of Medicine, Biodiscovery Institute, University of Nottingham, Nottingham, NG7 2RD UK; 2 Bucks Institute for Research on Aging, 8001 Redwood Blvd, Novato, CA 94945 USA; 3 Faculty of Medicine and Health Sciences, Queen’s Medical Centre, University of Nottingham, University Park, Nottingham, NG7 2UH UK; 4 Stroke Trials Unit, Mental Health & Clinical Neuroscience, School of Medicine, University of Nottingham, Nottingham, NG7 2UH UK; 5 Academic Stroke, Mental Health & Clinical Neuroscience, School of Medicine, University of Nottingham, Nottingham, NG7 2UH UK

**Keywords:** Endothelial progenitor cells, Diabetes, Angiogenic regulators, Inflammation

## Abstract

Endothelial progenitor cells (EPCs) are stem cells that can repair injured blood vessels through neovascularisation. This is achieved through secretion of growth factors and endothelial maturation. EPC numbers and function have been studied to determine their diagnostic, prognostic and therapeutic potential in many ischaemic diseases such as stroke. However their activation homing and migration is not definitively understood in stroke patients. In this study, we profiled the non-stroke control group recruited into the Dunhill Medical Trust Endothelial Progenitor Cell Study. Demographic, clinical and plasma levels of angiogenic regulators of participants were analysed to determine if there was any correlation with EPC numbers, subtypes and function. Participants with diabetes had significantly supressed EPC numbers (CD45-CD34 + CD133 + KDR+) and CD34 + KDR + and KDR + EPC subtypes. Male participants had significantly lower EPC numbers compared to female participants and the proliferative capacity of endothelial colony forming cells significantly decreased with increasing participant age. Pro-angiogenic proteins such as granulocyte colony-stimulating factor and stromal cell-derived factor were positively correlated with both undifferentiated and endothelial-committed EPC subtype numbers (CD133+, KDR+, CD34 + CD133+, CD34 + KDR+), whereas anti-angiogenic proteins such as thrombospondin-1 showed a negative correlation with undifferentiated EPC subtypes (CD133+, CD34 + CD133+) but a positive correlation with endothelial-committed EPC subtype numbers (KDR+, CD34 + KDR+). These results show that EPC numbers and subtypes are affected by many factors and larger studies which can analyse and deconvolute the interactions between comorbidities, plasma biomarker levels and EPC are needed.

**Graphical Abstract:**

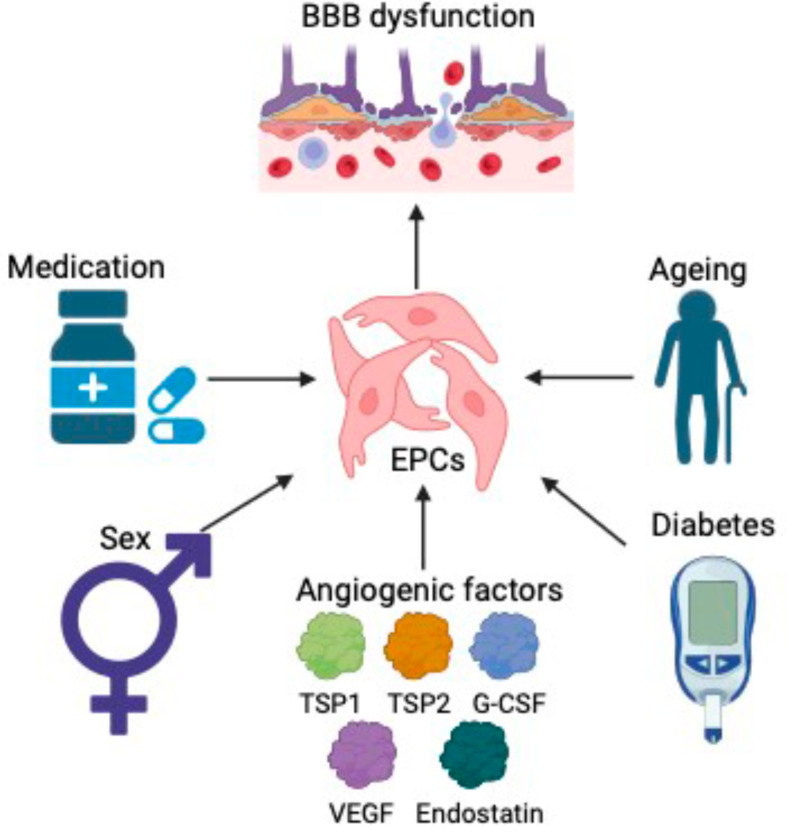

## Introduction

Endothelial progenitor cells (EPCs), a type of stem cell, have long been studied as an avenue of therapeutic intervention in many diseases with vascular injury such as stroke, heart disease and hypertension. EPCs are recruited to the site of injury and can repair damaged vessels through promotion of angiogenesis and vasculogenesis, and differentiation into mature endothelial cells [1–3].

Studies have investigated their potential as diagnostic and prognostic markers for stroke [4] and cardiovascular disease [5]. However, these studies along with clinical trials have given mixed results [6]. The complex landscape of potential factors that affect EPC numbers and functionality may contribute widely to the differences in results seen. Inflammation, angiogenic activators and inhibitors, health conditions and medications such as statins have all been shown to affect EPC numbers and, to a certain extent, function [7].

This field is further complicated by the lack of unique markers for the identification of EPCs. There is no consensus between researchers, and usually a combination of the following 3 or 4 markers, CD45, CD34, CD133 and KDR are used. More recent studies have extensively phenotyped circulating progenitor cells with the aim to fully characterise EPCs and indicate markers such as CD19 may be more appropriate in defining EPCs [8]. In a similar context, efforts to standardise methods employed to quantify and culture peripheral blood-derived endothelial colony forming cells (ECFCs), a functional subtype of EPCs, indicate that ECFC levels should be expressed as the number of colonies per 10^7^ mononuclear cells (MNCs) seeded [9].

This exploratory study aimed to profile the healthy volunteer control group from the Dunhill Medical Trust Endothelial Progenitor Cell (DMT-EPC) study [10] to investigate whether any clinical or demographic features or blood biochemical analytes correlated with overall EPC numbers (CD45-CD34 + CD133 + KDR+) or certain EPC subtypes.

## Methods

### Study Population

Data from 90 healthy volunteers recruited into the DMT-EPC study (NCT02980354) were used in the current paper. The DMT-EPC study was reviewed and approved by West Midlands Coventry & Warwickshire Research Ethics Committee (16/WM/0304). The details of the study design and recruitment have previously been described [10]. Briefly, 30 mL of peripheral blood was taken from the healthy volunteers and used for multiple assays: quantification of the number of circulating endothelial progenitor cells; measurement of the plasms biochemical profile; and culture of the EPCs to obtain endothelial colony-forming cells (ECFC). Individuals with ≥ 140/90 mmHg blood pressure, ≥ 5 mmol/L total cholesterol level, ≥ 7 mmol/L fasting glucose or 11.1 mmol/L 2 h post-prandial glucose level were respectively considered as hypertensive, hyperlipidaemic, and diabetic. Demographic and clinical characteristics for the individuals are shown in Table 1.

**Table 1:** Clinical and demographic features of the participants involved in the current study

Number of participants	90
Age /median (IQR)	61.5 (34–69.75)
Sex /male (%)	35 (39)
Hypertension /yes (%)	21 (23)
Hyperlipidaemia /yes (%)	15 (17)
Diabetes mellitus /yes (%)	9 (10)
Coronary artery disease /yes (%)	2 (2)
Deep vein thrombosis /yes (%)	1 (1)
Statins /yes (%)	21 (23)
ACE inhibitor /yes (%)	16 (18)
Calcium channel blocker /yes (%)	8 (9)
Antiplatelets /yes (%)	7 (8)
Anticoagulants /yes (%)	2 (2)
Diuretics /yes (%)	6 (7)
Glucose lowering /yes (%)	6 (7)
Nitrates /yes (%)	2 (2)
Insulin /yes (%)	1 (1)

*None of the participants had a Transient ischaemic attack, Ischaemic stroke, Intracerebral haemorrhage or Peripheral arterial disease

### Measurement of EPC Numbers Using flow Cytometry

Blood was diluted in phosphate buffered saline then loaded onto Histopaque 1077 (Sigma, Dorset, UK) and centrifuged. The buffy coat layer was stained with conjugated antibodies against CD45 (FITC, BD Biosciences, Berkshire, UK), CD133 (APC, Miltenyi Biotech, Surrey, UK), CD34 (APC-Cy7, BD Biosciences, Berkshire, UK) and KDR (PE, R&D Systems, Abingdon, UK) and 1 million events were counted using a BD FACS Canto II flow cytometer (BD Biosciences, Berkshire, UK). Data were processed and the number of non-haematopoietic cells (CD45-) expressing markers for immaturity (CD133), stemness (CD34), and/or endothelial maturity (KDR) were counted as previously described [4] and considered to be EPCs.

### Culture of ECFC

Blood was processed as above. The buffy coat was seeded on fibronectin-coated well plates in EBM-2 Medium (Lonza, Cambridge, UK) supplemented with 20% foetal bovine serum (Sigma, Dorset, UK) to acquire the ECFC as previously described [11].

### Measurement of Plasma Biochemical Profile

Blood was centrifuged to obtain the plasma which was aliquoted and stored at -80 °C until analysis. ELISAs to measure the levels of TNF-α, G-CSF, PDGF-BB, SDF-1, VEGF, thrombospondin-1, thrombospondin-2 and endostatin were purchased from R&D Systems (Abingdon, UK). An ELISA to measure the levels of angiostatin was purchased from Abcam (Cambridge, UK). All kits were used according to the manufacturers’ instructions.

### Measurement of Total Antioxidant Capacity

Blood was centrifuged to obtain the plasma which was aliquoted and stored at -80 °C until analysis. An assay to measure the total anti-oxidant capacity was purchased from Abcam (Cambridge, UK). Briefly, plasma was mixed with the copper working reagent, incubated for 90 min on an orbital shaker at room temperature after which the absorbance was measured at 570 nm. A Trolox standard curve, provided with the kit, was used to determine total antioxidant capacity of the plasma sample.

### Proliferative Capacity

The 4-[3-(4-lodophenyl)-2-(4-nitrophenyl)-2 H-5-tetrazolio]-1.3-benzene disulfonate (WST-1) kit (Roche, Hertfordshire, UK) was used to measure the proliferative capacity of ECFC. ECFC were cultured in 96-well plates and after 48 h, the media was replaced with fresh medium containing 10% WST-1. The plates were incubated for 2 h at 37 °C before the absorbance at 450 nm was measured using a FLUOstar Omega plate reader (BMG Labtech, Aylesbury, UK).

### Migratory Capacity

ECFC were incubated in EBM-2 media supplemented with 3.5 mg/ml bovine serum albumin (Sigma, Dorset, UK) and 5 µg/ml Calcein-AM (Molecular Probes, Loughborough, UK) for 2 h then seeded into 3 μm pore HTS FluoroBlok inserts (BD Falcon, Flintshire, UK) before assessing their migration across the insert into the above media containing 50 ng/ml VEGF. Readings were taken from the bottom of the plate using a FLUOstar Omega plate reader (BMG Labtech, Aylesbury, UK)).

### Statistical Analysis

Statistical analyses were conducted using GraphPad Prism 8.4.3 (GraphPad Software Inc.), SPSS version 26 (SPSS Inc.) or R packages (MASS, lmtest, gmodels). Data were tested for normality using the Shapiro Wilk normality test. Statistical tests were performed using t-test, Mann-Whitney, Pearson or Spearman correlation analysis and negative binomial regression where appropriate. *P* < 0.05 was considered as significant.

## Results

### Clinical and Demographic Features

The 90 control participants had a median age 61.5 years and consisted of 55 (61%) females. The presence of health conditions and medication use was relatively low, with hypertension (23%) and statin use (23%) being the most common (Table 1).

### Health Conditions Increase TNF-α, SDF-1 and VEGF Levels

No differences were seen in any of the plasma markers (TNF-α, G-CSF, PDGF-BB, SDF-1, VEGF, thrombospondin-1, thrombospondin-2, endostatin, angiostatin and total antioxidant capacity) between male and female participants. TNF-α levels were significantly increased in participants who had hypertension, diabetes, hyperlipidaemia and/or were taking statins or calcium channel blockers. SDF-1 levels were significantly increased in participants who had diabetes. VEGF levels were significantly increased in participants who had hyperlipidaemia and/or were taking calcium channel blockers (Table 2).

**Table 2:** P-value results from the statistical analysis comparing the blood biomarkers and endothelial progenitor cell subtypes between male and female groups and between participants with and without clinical features or medications. For the plasma biomarkers, the p values for the Mann Whitney U-Test analyses are shown. Where the p value is < 0.05 the ratio of biomarker level means between participants with: without comorbidity/medication is given in brackets. For the endothelial progenitor cell subtypes, the presence of a single comorbidity or use of medication was assessed. P values from the negative binomial model analyses are shown. Age and sex of participants were included in all models. Where the p value is < 0.05 the exponentiated values of the coefficients estimate and confidence intervals are shown in round and square brackets respectively

PLASMA BIOMARKERS							
BIOMARKERS	Sex (M vs. F)	Hypertension	Diabetes Mellitus	Hyperlipidaemia	Statins	ACE inhibitor	CCB
TAC	0.742	0.239	0.207	0.987	0.914	0.800	0.700
TNF-α	0.368	**0.023** **(3.25)**	**0.006** **(3.75)**	**0.027** **(1.24)**	**0.022** **(1.18)**	0.277	**0.029** **(1.36)**
G-CSF	0.341	0.171	0.551	0.379	0.554	0.328	0.628
SDF-1	0.223	0.711	**0.026** **(2.99)**	0.891	0.501	0.918	0.139
VEGF	0.791	0.091	0.357	**0.030** **(1.17)**	0.074	0.090	**0.030** **(1.25)**
PDGF-BB	0.154	0.053	0.507	0.656	0.377	0.219	0.744
Thrombospondin-1	0.135	0.073	0.234	0.644	0.562	0.727	0.898
Thrombospondin-2	0.104	0.398	0.383	0.577	0.676	0.982	0.317
Angiostatin	0.081	0.370	0.654	0.416	0.968	0.809	0.921
Endostatin	0.872	0.338	0.559	0.360	0.123	0.627	0.419
**ENDOTHELIAL PROGENITOR CELL SUBTYPES**							
SUBTYPES	Age + Sex	Hypertension	Diabetes Mellitus	Hyperlipidaemia	Statins	ACE inhibitor	CCB
CD34+	**Male 0.0396** **(0.513)** **[0.263–1.02]** Age 0.473	0.230	0.119	**0.00479** **(3.26)** **[1.36–8.45]**	**0.0259** **(2.39)** **[1.01–5.73]**	**0.000203** **(4.62)** **[1.97–11.8]**	0.0790
CD133+	Male 0.803 Age 0.0816	0.536	0.989	0.454	0.483	**0.0172** **(3.15)** **[1.30–8.85]**	0.637
KDR+	Male 0.381 Age 0.623	0.173	**0.0483** **(0.175)** **[0.0348-1.69]**	0.660	0.605	0.087	0.0905
CD34 + CD133+	Male 0.402 Age 0.0664	0.680	0.967	0.459	0.674	**0.0263** **(2.92)** **[1.20–8.21]**	0.334
CD34 + KDR+	Male 0.560 Age 0.571	**0.0367** **(4.03)** **[0.909–19.6]**	**8.7 × 10** ^**− 07**^ **(0.0124)** **[0.00289-0.114]**	0.914	0.595	**0.0123** **(5.86)** **[1.54–29.8]**	**0.00218** **(0.0660)** **[0.0130–0.688]**
CD133 + KDR+	Male 0.217 Age 0.0797	0.242	0.471	0.954	0.652	0.119	0.595
CD34 + CD133 + KDR+	Male 0.357 Age 0.0961	0.250	**0.0297** **(0.123)** **[0.0219-1.54]**	0.643	0.833	0.0901	0.147

*CCB*, Calcium channel blocker; *G-CSF*, granulocyte-colony stimulating factor; *PDGF-BB*, platelet-derived growth factor-BB; *SDF-1*, stromal cell-derived factor-1; *TAC*, total anti-oxidant capacity; *TNF-α*, tumour necrosis factor-α; *VEGF*, vascular endothelial growth factor

### Diabetes Suppresses the Total Number of EPCs

From the negative binomial regression results, diabetes was the only health condition to affect EPC numbers (cells which were negative for CD45 and positive for CD34, CD133 and KDR). Having diabetes was shown to be associated with a significant decrease in the number of EPCs compared to participants without diabetes. No other health condition or medication was shown to affect total EPC numbers. Diabetes was also shown to be significantly associated with a reduction in the number of CD34 + KDR + and KDR + EPC subtypes.

### EPC Subtype Numbers are Increased in Females and Participants with Hypertension and Hyperlipidaemia

Male participants had a significant 48.7% reduction in the CD34 + EPC subtypes compared to females. Participants with hypertension had 4-fold increase in CD34 + KDR + EPC subtype numbers compared to those without. Similarly, participants with hyperlipidaemia had 3.26-fold increase in CD34 + EPC subtype numbers. Statins also significantly increased EPC numbers by 2.39-fold. Participants taking ACE inhibitors saw the largest increase in EPC subtypes with significant increases in CD34+, CD133+, CD34 + CD133 and CD34 + KDR + subtypes. Participants taking calcium channel blockers also had significantly reduced CD34 + KDR + EPC subtypes. (Table 2).

### Stratification of EPC Number According to Age and Plasma Biomarker Level Revealed General Trends

**Fig. 1:**
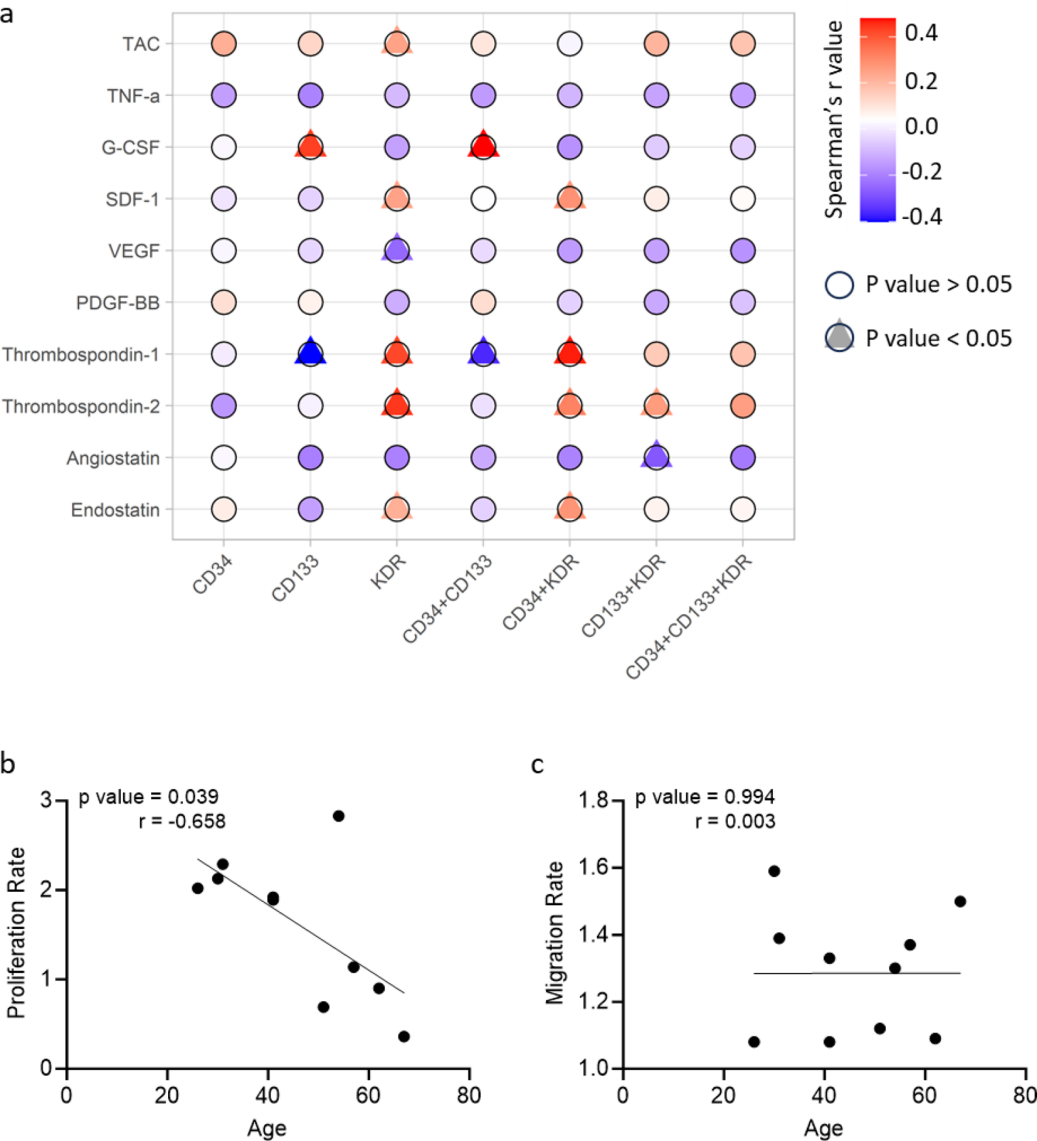
Plasma biomarker, endothelial progenitor cell subtype and functional correlations. (**A**) The blood biochemical markers were correlated to endothelial progenitor cell subtypes using Spearman’s rank correlation. The bubble grid shows these correlations with the colour indicative of the r value and the shape indicative of p value. The proliferation (**B**) and migration (**C**) rates were correlated with participant age. Functional properties of outgrowth endothelial progenitor proliferation rate of participants using Pearson correlation. *P* < 0.05 was considered statistically significant in all analyses. G-CSF, granulocyte-colony stimulating factor; PDGF-BB, platelet-derived growth factor-BB; SDF-1, stromal cell-derived factor-1; TAC, total anti-oxidant capacity; TNF-α, tumour necrosis factor-α; VEGF, vascular endothelial growth factor

The number of comorbidities and medications was shown to increase with age. A positive trend between EPC number and total antioxidant capacity and thrombospondin-1 and − 2 levels and a negative trend between EPC number and VEGF, angiostatin and TNF-α levels was observed (Fig. 1).

### EPC Subtype Numbers Correlate with Angiogenic Regulators

**Fig. 2:**
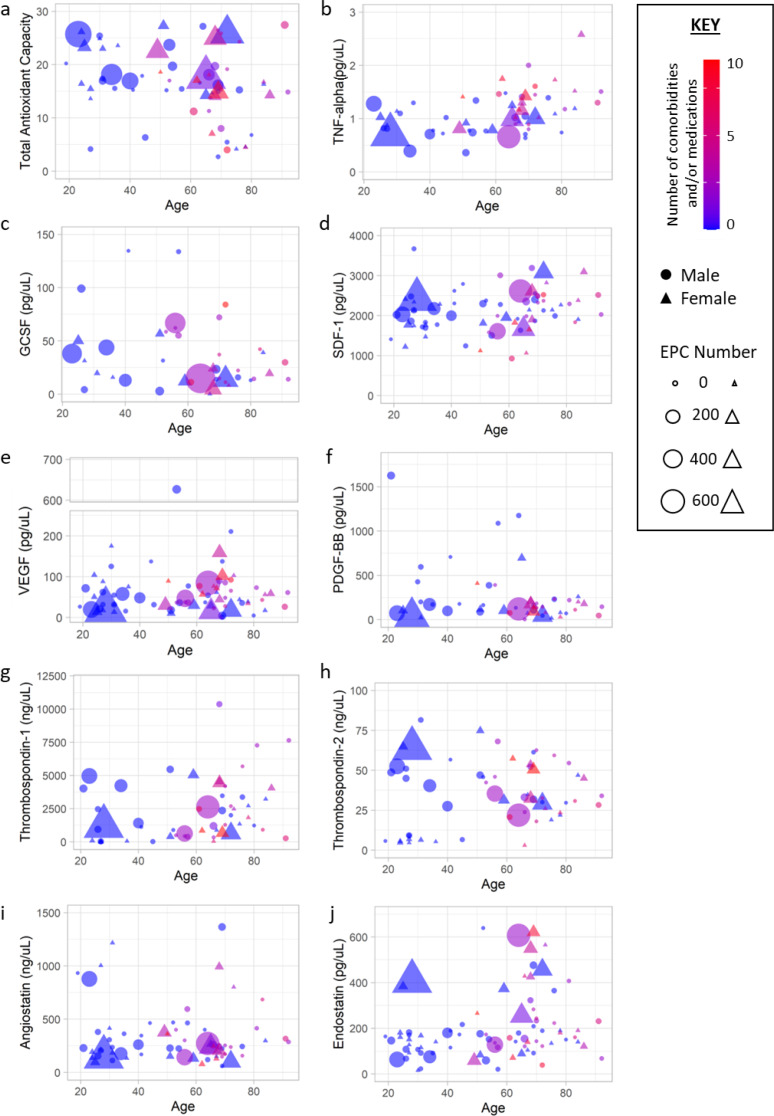
Participant comorbidity, medication and endothelial progenitor cell number spread according stratified by plasma biomarkers. (**A**-**J**) The bubble plots show 5 pieces of information. Plasma biomarker levels are on the y-axis and age on the x-axis. The shape indicates whether the participant is male (circle) or female (triangle). The size of the shape indicates the number of endothelial progenitor cells, with a bigger area indicating more cells. The colour of the shape indicates the number of comorbidities and/or medication the participant has, from zero (blue) to ten (red). Comorbidities include hypertension, diabetes, atrial fibrillation, hyperlipidaemia, coronary artery disease and deep vein thrombosis. Medications include statins, ACE inhibitors, calcium channel blockers, anticoagulants, antiplatelets, nitrates, diuretics, insulin and glucose lowering

CD133 + and CD34 + CD133 + cell numbers were significantly positively correlated with G-CSF and negatively correlated with thrombospondin-1. The numbers of KDR + cells were significantly positively correlated with total antioxidant capacity, SDF-1 and thrombospondin-1 and − 2 levels. Contrastingly, these EPC subtypes were significantly negatively correlated with VEGF levels. In addition, the numbers of CD34 + and KDR + cells were also positively correlated with endostatin levels, whereas cells positive for CD133 and KDR were negatively correlated with angiostatin levels (Fig. 2a).

### Proliferative Capacity of ECFC Decreases with Age

The proliferation rate of ECFC significantly decreased with increasing participant age. However, the migration rate did not correlate with age (Fig. 2b-c).

## Discussion

The number of ECFC colonies and relevant EPC counts as well as the phenotypic and functional characteristics of early EPCs and ECFCs were documented in previous studies [4, 12]. Despite decreases in individual and collective numbers of EPC subtypes expressing CD34, CD133 and/or KDR antigens in elderly population [11], rates of successful ECFC isolation was reported to be similar for individuals aged 20–73 years [9]. In light of these findings, this exploratory study investigated whether any clinical, demographic or plasma biochemical features from participants correlated with circulating EPC numbers. We showed that: diabetes is associated with lower EPC numbers; and the levels of anti-angiogenic factors such as thrombospondin-1 and − 2 and pro-angiogenic factors such as G-CSF and SDF-1 are correlated with EPC subtypes.

In the current study, participants with hypertension, hyperlipidaemia, diabetes, and history of statin and calcium channel blocker use had significantly higher TNF-α levels compared to participants without these health conditions indicating an increased level of inflammation. TNF-α, an important inflammatory mediator following injury such as tissue ischaemia [13] has been shown in vitro to increase EPC migration [14] but to also reduce EPC number [15]. However, the effect of inflammation on EPC numbers and functionality is complicated and dependent on the stimuli [16]. These patient studies are confounded by comorbidities and the study of TNF-α in isolation does not represent the complicated in vivo environment. For example, in this study, in the same participants, the total antioxidant capacity remained unchanged, indicating that there may be some impairment of endogenous mechanisms to reduce inflammation [17]. Total antioxidant capacity was positively correlated with KDR + endothelial-committed EPC numbers indicating higher levels of endogenous antioxidants may increase stem cell endothelial maturity and may be a possible avenue for future therapeutic research in these health conditions.

Interestingly, although the level of chemokine SDF-1 was shown to be almost ~ 3-fold higher in participants with diabetes, as reported in the literature [18], the number of CD34 + CD133 + KDR + and KDR + cells were substantially suppressed. Diabetes has been shown to impair EPC number and function in previous studies [19, 20] and taken together these findings may indicate that the endogenous vasculo-protective/repair system is profoundly impaired in this condition. Furthermore, the increase in SDF-1 levels may be a stress response to this impaired system, and an attempt to try and increase the recruitment and mobilisation of EPCs.

Conversely, when looking at hypertension, endothelial-committed CD34 + KDR + cells were significantly increased (~ 4 fold) and participants taking ACE inhibitors also showed an increase (~ 3–6 fold) in both undifferentiated (CD34+, CD133+, CD34 + CD133+) and endothelial-committed (CD34 + KDR+) cell numbers. A previous study has shown CD34 + and CD34 + KDR + cell numbers to be reduced in poorly controlled hypertensive patients [21], unfortunately, it is not possible to ascertain whether the participants in our study had poorly controlled hypertension and therefore this would need further investigation. Studies have shown conflicting findings on the effect of ACE inhibitors on EPC numbers in many diseases such as diabetes and stroke [22–24]. Results from our study would indicate that hypertension increases stem cell maturity to form endothelial cells and ACE inhibitors may also have an effect of increasing stem cell numbers and maturation. The effect of multiple comorbidities and medications adds a layer of complexity in defining their effect on EPCs and more realistically the EPCs will be affected by multiple pathways.

Further investigation into both pro- and anti-angiogenic factors showed correlation with the number of circulating EPC subtypes. Angiogenic modulators showed a discrete pattern in regulating the level of EPCs subtypes. For instance, while thrombospondin-1 negatively correlated with undifferentiated stem cells (CD133+, CD34 + CD133+), this anti-angiogenic factor was positively correlated with endothelial-committed cells (KDR+, CD34 + KDR+). These positive correlations were somewhat mirrored by thrombospondin-2 and endostatin. This duality could be attributed to the fragments of thrombospondin-1 which have both pro- and anti-angiogenic properties [25]. These results indicate that in participants with higher levels of these anti-angiogenic markers there may be a mechanism by which the undifferentiated EPC subtype population is reduced which may have a compensatory effect of increasing the endothelial maturation of the existing stem cells.

When investigating the correlations of the pro-angiogenic markers we found G-CSF showed a strong positive correlation to undifferentiated stem cells (CD133+, CD34 + CD133+) and SDF-1 was positively correlated to endothelial-committed cells (KDR+, CD34+/KDR+). These results are unsurprising. Many studies have shown G-CSF mobilises CD34 + and CD133 + cells [26, 27] and clinical trials have investigated its therapeutic use in diseases such as cancer, stroke and liver failure, with mixed results [28–30]. SDF-1 has also been shown to increase EPC recruitment [31]. The results from this study indicate higher levels of these pro-angiogenic markers results in greater EPC numbers and could be beneficial in vascular diseases. However, these markers do not operate independently. Therefore, further research is required with a larger cohort of participants to look at the interaction between these factors to fully determine their effect on EPC numbers.

As well as EPC numbers, their functionality is also a key factor in tissue repair [32]. In this study, participant age had no effect on the number of circulating EPCs. However, a strong negative correlation between ageing and the proliferation rate of ECFC was found, indicating that advancement of age is associated with lower proliferative ability, as has been previously reported [33].

This exploratory study has revealed novel avenues for future research, especially with regards to EPC subtypes and angiogenic regulators. This study has shown a strong link between diabetes and EPC numbers. The association between pro-and anti-angiogenic chemokines and EPC subtypes, which has not been shown beforewarrants further investigation to uncover potential therapeutic targets.

### Limitations

This current study lacked the power to analyse multiple predictor variables in the regression analysis, therefore a replication cohort with a larger study population would increase confidence in these results be able to evaluate the complexity of multiple health conditions and medications especially between male and female participants [34]. In the current study, severity of disease was not evaluated. This is important in diseases such as diabetes and hypertension and should be considered in future studies.

## Data Availability

The datasets generated during and/or analysed during the current study are available from the corresponding author on reasonable request.
